# Resilience and Post-traumatic Growth among Cancer Patients: A Moderated Mediation Analysis through Perceived Social Support and Stress

**DOI:** 10.11621/pir.2024.0203

**Published:** 2024-06-30

**Authors:** Kaneez Zahra, Saira Khan, Rayna Sadia, Irum Aslam

**Affiliations:** a *National Institute of Psychology, Quaid-i-Azam University, Islamabad, Pakistan*; b *Riphah International University, Gullberg Greens Campus, Islamabad, Pakistan*; c *Rawalpindi Women University, Rawalpindi, Pakistan*

**Keywords:** cancer, Post-Traumatic Growth (PTG), stress, resilience, social support

## Abstract

**Background.:**

A cancer diagnosis is a powerful, unanticipated, and occasionally traumatic event which impacts an individual with evidence of a life-threatening illness. As a potentially terminal illness, cancer entails substantial physical, emotional, and psychological costs. Even though psychological resources such as social support and resilience promote post-traumatic growth, chronic stressors experienced by cancer patients have the potential to weaken the function of such positive resources. Therefore, it is crucial to assess how stress impacts post-traumatic growth among cancer patients.

**Objective.:**

The present study aimed to examine the moderating effect of stress on post-traumatic growth and resilience among cancer patients mediated by perceived social support.

**Design.:**

A cross-sectional research design and purposive sampling technique was used to collect data on Urdu versions of the Short Form of the Post-traumatic Growth Inventory, Brief Resilience Scale, Multidimensional Scale of Perceived Social Support, and Stress subscale of the Depression, Anxiety, Stress Scale. Cancer patients (*N* = 200) were approached and recruited from public and private hospitals in Rawalpindi, Islamabad, and Gilgit Baltistan to participate in the research.

**Results.:**

Post-traumatic growth had a positive association with resilience and perceived social support. However, stress was negatively related to all study variables. Moderated mediation analysis highlighted that high levels of stress decrease the indirect impact of resilience on post-traumatic growth through perceived social support.

**Conclusion.:**

The study’s findings imply that stress must be given considerable attention while fostering post-traumatic growth among cancer patients. Based on these findings, future studies should also take into account specific age range of the sample, types of cancer (and other terminal illnesses), the cross-sectional nature of the study, and individual differences in coping with illness for a comprehensive understanding of post-traumatic growth among cancer patients.

## Introduction

Cancer, a potentially terminal illness caused by the mutation of DNA, is among the leading causes of death across the globe. Growing issues related to rapid aging populations (Altunal & Şahiner, 2022; Khan et al., 2023), increasing numbers of inactive lifestyles (Yang et al., 2023), unhealthy lifestyles (Cusinato et al., 2020), and air pollution (Gu et al., 2023), pose a threat of more cancer cases. Excluding non-melanoma skin cancer, which accounted for 18.1 million cases, approximately 10 million people of the projected 19.3 million new cases of cancer in 2020 died from the disease (Merrigan et al., 2023).

Recent evidence suggests that the interplay between genetic and environmental factors contributes to the development of cancer (Boyce et al., 2021; Ugai et al., 2022). Genetic variables such as alleles, chromosome number, or location, Single Nucleotide Polymorphisms (SNPs), and RS number interact with environmental factors such as exposure to arsenic, benzene, polychlorinated biphenyl (PCB), polycyclic aromatic hydrocarbons (PAHs), chlorinated dioxin, etc. Modifying factors involving poor diet, smoking, physical inactivity, and other lifestyle factors also impact the risk of developing cancer (Boyle, 2020; Mbemi et al., 2020).

Pakistan has 118,442 deaths from cancer and an estimated 178,388 new cancer cases per year (Ayub et al., 2022). Furthermore, the country exhibits the highest regional breast cancer incidence and mortality rate in Asia (Rashid et al., 2021), with one in every nine women currently at a lifetime risk of developing breast cancer (Sarwar et al., 2018). In Pakistan, oral cancer ranks second in terms of prevalence and is more common among men (15.9%) (Anwar et al., 2020). Lung, lips, mouth, and intestinal cancer account for most adult cases, regardless of gender (Niaz et al., 2017).

Despite the alarming prevalence of the disease, cancer patients remain at a disadvantage in Pakistan in terms of screening and treatment; this is due to lack of awareness (Hirani et al., 2021), poor socioeconomic status (Shamsi et al., 2020), the poor availability and affordability of cancer medicine (Sawrar et al., 2018), and limited treatment options. In addition, the Shaukat Khanum Memorial Cancer Hospital is the only hospital providing free treatment to cancer patients, but due to its limited capacity, only some patients can get accommodated there. Thus, in addition to the burden of the disease itself, cancer patients in Pakistan have to deal with numerous additional stressors that collectively exert a detrimental impact on their well-being and illness outcomes.

A cancer diagnosis is a powerful, unanticipated, and occasionally terrifying experience that affects the person and provides evidence of a potentially fatal illness (Cao et al., 2018; Gori et al., 2021). The crucial stages of diagnosis and treatment influence both the physical and mental functioning of the patient (Harrington & Venta, 2020). Whether or not the experience of receiving a diagnosis turns into a trauma depends on several factors, such as the prognosis, any direct or indirect experiences the patient may have previously had with cancer, available treatment options, and the patient’s personal coping resources, including support from family and friends (Fabi et al., 2020). Empirical findings imply an association between cancer and trauma with typical short- and long-term consequences (Harmon & Venta, 2021; Liu et al., 2020).

Studies have demonstrated that, following a cancer diagnosis and treatment, patients may report positive improvements in addition to psychological distress (Tu et al., 2020l; Wolfson et al., 2020). Broadly defined, Post-Traumatic Growth (PTG) is a beneficial psychological shift brought on by overcoming extremely difficult life situations. These constructive adjustments can be broadly divided into three distinct domains: changes in life philosophy, interpersonal connections, and self-perception. PTG is commonly identified in cancer patients, and accounts for 60% to 95% of cases (Peng et al., 2019). Numerous cancer survivors perceive personal gains from their illness, including better quality of relationships, a more profound understanding of life, and a more positive view of themselves. However, PTG is influenced by a variety of internal and external factors, including levels of social support (Cao et al., 2018), resilience (Zhang et al., 2019), and stress (Oh et al., 2021).

PTG is often described as a form of resilience (Atay Turan et al., 2023; O’Briien & Taku, 2023). In the context of cancer, resilience is characterized as a person’s adaptive characteristics and/or personal attributes that enable an effective adjustment of the disease. Optimism, positive emotions, self-worth, self-efficacy, cognitive flexibility, coping mechanisms, social support, and spirituality are a few indications of such traits (Seiler & Jeewein, 2019). Active interventions based on resilience might be a favorable option for cancer patients because they are based on characteristics that promote positivity (Festerling, 2023).

Cancer patients with extensive social support networks possess a greater ability to manage the demanding nature of their treatment, to maintain a positive outlook, and to achieve a better sense of self and life (Yang et al., 2023). Perceived social support is described as the conviction that supportive behaviors that come naturally—such as love, care, and attachment—are given when needed, usually by family, friends, or other important sources of support (Alfasi, 2023). Tedeschi and Calhoun’s (2004) social-cognitive processing model emphasized that children and adolescents’ cognitive adaptation and successful confrontation of psychological difficulties following a traumatic event can be facilitated by perceived social support, which ultimately fosters PTG. Empirical evidence confirms the favorable association between perceived social support, resilience, and PTG (Gu et al., 2023; Ning et al., 2023; Pak et al., 2020).

Stress is characterized as an individual’s mental, physical, and/or psychological response to stressors in his or her environment (Lu et al., 2021). Cancer-related stressors, in contrast to acute traumatic events with clear onsets and terminations, are complex, ongoing, and difficult to pinpoint (for example, ongoing threats, fear of recurrence, and prospective worries) (Tometich et al., 2020). Even if cancer patients have an abundance of positive personal attributes, the external and internal stressors they encountered tend to exert a detrimental impact on their illness outcomes (Lee et al., 2023). The routines and responsibilities of daily life, such as work, family, and finances, can be common sources of stress. External factors such as poor living conditions, exposure to unhealthy environmental conditions, poverty, discrimination, and inequalities in the social determinants of health are additional stressors. Studies conducted with varying samples show that high levels of stress are negatively associated with positive attributes such as resilience and social support (Cusinato et al., 2020; Haynen et al., 2020; Yalçın et al., 2022).

According to the Transactional Model of Stress and Coping (Folkman & Lazarus, 1984), transactions (or interactions) between a person and their environment have an impact on a person’s ability to deal with challenges and eventually adapt to them. In the context of cancer patients in Pakistan, the level of pre-existing disease-specific distress may be exacerbated by financial and treatment-related challenges. Subsequently, the ability to effectively cope with the disease and grow past the trauma may be altered even in the presence of coping and personal resources (such as social support and resilience).

The Life Crisis and Personal Growth Model developed by Schaefer and Moos (1992) posits that a person’s internal systems and external resources collaborate to impact event-related factors when a crisis takes place. The individual’s coping strategies and cognitive evaluation are altered as a result, resulting in a positive outcome. The interdependence of external resources and human systems directly influences positive results. Based on this theory, patients with cancer combine their personal systems (resilience) and external resources (social support) to achieve positive outcomes (PTG).

**Figure 1.**
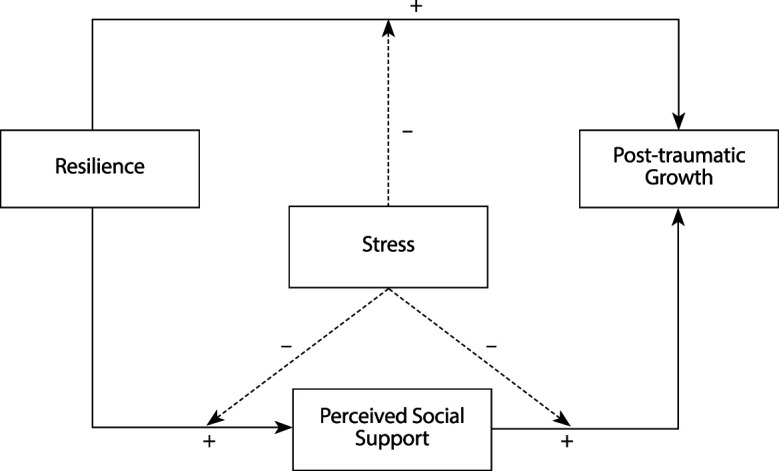
Conceptual Framework of the Study

However, because the diagnosis of cancer is a major stressor itself and is accompanied by various other stressors (including financial concerns, lack of access to treatment in developing countries like Pakistan, death anxiety, and disease-related distress), the impact of positive sources such as perceived social support and resilience on post-traumatic growth may be affected. The present study, therefore, proposed a moderated mediation model to examine the moderating effect of stress on the association between resilience and post-traumatic growth mediated by perceived social support. The conceptual model of the study is presented in *Figure 1.*

### Hypothesis of the Study

Based on the literature, we hypothesized that stress moderates the indirect effect of resilience on post-traumatic growth among cancer patients mediated by perceived social support.

## Method

### Participants

The study was cross-sectional and correlational in nature, and the purposive sampling technique was used to collect the data. The eligibility criteria for inclusion in the study were : 1) patients clinically diagnosed with any type of cancer; and 2) cancer patients with the ability to read and write. Patients living with any other terminal disease, or a psychiatric illness, were excluded from the study.

In the study group (*N* = 200), the age of participants ranged between 18 and 100 years (*M* = 50.30, *SD* = 14.26). The percentage of male and female participants was roughly equal (48% and 52% respectively), and the large majority were married (90%). The majority of participants were graduates (42%), followed by the uneducated (31%) and post-graduates (27%). More than half of the participants were residing in extended family situations (57.5%). Regarding their specific cancer profiles, some participants had a history of a one-time relapse (12.3%). The duration of cancer for most of the participants was 1–3 years (88%) while the most common stage of cancer was stage 2 (38%). The commonly utilized methods of treatment included chemotherapy (87.7%), surgery (52%), and radiotherapy (51.5%). The frequently occurring comorbid illnesses included hypertension (23%) and diabetes (19.1%). Additionally, many participants were seeking treatment from government hospitals (69%). Detailed demographic characteristics of the sample are presented in *Table 1*.

**Table 1:** Demographic Characteristics of the Sample (N = 200)

**Variable**	*n (f)*
Gender	
Male	96 (48%)
Female	104 (52%)
Education	
Graduates	84 (42%)
Postgraduates	54 (27%)
Uneducated	62 (31%)
Marital Status	
Married	180 (90%)
Unmarried	20 (10%)
Family System	
Nuclear	85 (42.5%)
Joint	115 (57.5%)
Family History of Cancer	
Yes	68(34%)
No	132(66%)
History of Relapse	25(12.3%)
Hospital Type	
Government	138(69%)
Private	62(31%)
Duration of Cancer	
1–3 years	176 (88%)
4 years or more	24 (12%)
Stage of Cancer	
Stage 1	44 (22%)
Stage 2	76 (38%)
Stage 3	38 (19%)
Stage 4	42 (21%)
Mode of Treatment	
Surgery	106 (52.0%)
Chemotherapy	179 (87.7%)
Radiotherapy	105 (51.5%)
Bone Marrow Transplant	1 (.05%)
Immunotherapy	4 (2%)
Comorbid Illnesses	
Diabetes	*39(19.1%)*
Hypertension	47(23%)
Obesity	9(4.4%)
Other Complications	1(.05%)

### Procedure

Considering the convenience of the researcher and potential diffculties that may arise while approaching cancer patients, 200 cancer patients were included in the study. The patients were personally approached by the researcher, who visited various public and private hospitals in Islamabad, Rawalpindi, and Gilgit Baltistan. The data collection was carried out between January and June 2023. The study plan was approved by the Ethical Committee of the Quaid-i-Azam University, Islamabad.

### Questionnaires

Self-report measures were employed in the operationalization of the study variables. Considering the diverse nature of the sample, Urdu versions of the scales were used in the study. All the scales were translated by their respective authors using the forward and backward translation approach and found to be empirically valid and reliable measures of their respective constructs. Each scale is briefly described below.

***Sociodemographic Information*.** Sociodemographic and disease-related information was collected by the researcher.

***Brief Resilience Scale (BRS)*.** The 6-item BRS (Smith et al., 2008) was used to assess resilience. The scale was translated into Urdu language by the researcher before it was used in the present study. The construct validity and the factor structure of the translated scale was established through Confirmatory Factor Analysis (See *Table 2*). In the present study, the Cronbach’s reliability of the scale was .70.

***Short Form of the Post-Traumatic Growth Inventory (PTGI-SF)*.** Post-Traumatic Growth was assessed using the 10-item PTGI-SF (Cann et al., 2010). The Urdu-translated version of the scale was used (Aslam & Kamal, 2019). The scale assesses post-traumatic growth on a 6-point scale across five domains, including: 1) relationships with others; 2) realizing new possibilities in life; 3) perception of increased individual strength; 4) appreciation of life; and 5) spiritual change. In the present study, Cronbach’s alpha for the scale was .96.

***Multidimensional Perceived Social Support Scale (MPSS)*.** Perceived social support was assessed using the 12-item MPSS (Zimet et al., 1988). The Urdu version translated by Shahid (2010) and assessed stress on a 7-point scale. The MPSS comprises three subscales including family support, friend support, and support by others. A composite score is obtained after summing the scores for all items, with high scores indicating greater perceptions of social support. In the present study, Cronbach’s alpha for the scale was .96.

***Depression, Anxiety, Stress Scale (DASS).*** Stress was assessed using the 7-item stress subscale of the DASS (Lovibond & Lovibond, 1995). The current study utilized the Urdu version of the subscale translated by Aslam (2007). The subscale evaluates trouble in relaxing, nervousness, irritability, and agitation on a 4-point rating scale. The Cronbach’s alpha for the scale was .93 in the current study.

## Results

Confirmatory Factor Analysis (CFA) was conducted to validate the Brief Resilience Scale (Smith et al., 2008) in the Urdu language, determine its construct validity in the indigenous culture, and appraise its factor structure. The Analysis of Moment Structure (AMOS Graphic 26) was used for carrying out the CFA. The model was assessed using the Comparative Fit Index (CFI), the Tucker Lewis Index (TLI), the Incremental Fit Index (IFI), the Goodness of Fit Index (GFI), and the Root Mean Square Error of Approximation (RMSEA). According to the criteria set by numerous researchers (Dattalo, 2013; Hoyle & Isherwood, 2013), fit indices in the social sciences include the values of RMSEA, which are typically categorized and interpreted as follows: a close fit (.00 –.05); a fair fit (.05 –.08); a mediocre fit (.08 –.10); and a poor fit (over .10). The CFI, TLI, IFI and GFI should have values of .90 or higher.

**Figure 2.**
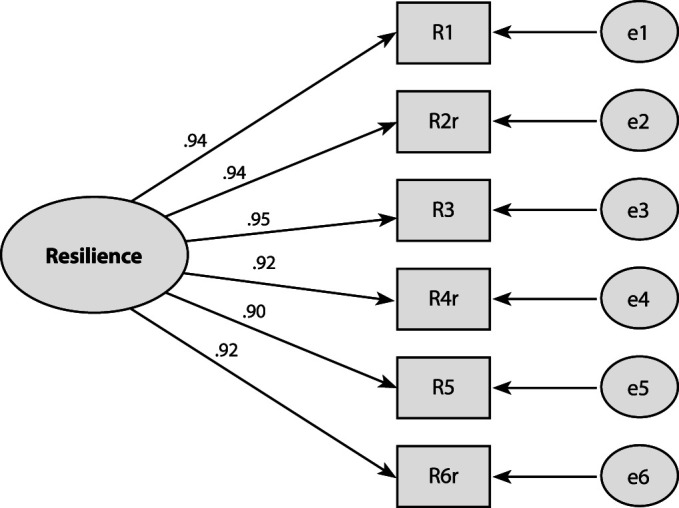
Measurement Model of Brief Resilience Scale

The results presented in *Table 2* show that the values of the fit indices demonstrated a good fit of the model to the observed data. The factor loadings of all items (as presented in the path diagram, *Figure 2*) ranged from .90 – .95. In conclusion, the fit indices and the factor loadings justified the factorial validity of translated Brief Resilience Scale (Smith et al., 2008).

**Table 2:** Model Fit Indices for Confirmatory Factor Analysis of the Brief Resilience Scale (N = 200)

Model	*X* ^2^	*df*	*p*	CMIN/*df*	Fit Indices				
					*CFI*	*GFI*	*TLI*	*IFI*	*RMSEA*
Model 1	15.22	9	.04	1.69	.99	.97	.99	.99	.05

The data was later analyzed using SPSS to compute the internal consistency reliabilities of the measures, and gauge the directional relationship among the study variables. The normality of the data was checked using descriptive statistics. Indices of skewness and kurtosis demonstrated normal distribution of the data, which were within the range of -2.69 to +2.96 as per the criteria given by Field (2009). Finally, the proposed moderated mediation model was tested using Process *Macro* (Model 59) (Hayes, 2013).

Correlation analysis was run to find the direction and trend in relationships among the study variables. The results showed that post-traumatic growth was positively associated with resilience (*r* = .68, *p* < .01) and perceived social support (*r* = .79, *p* < .01), but negatively associated with stress (*r* = -.55, *p* < .01). Stress was found to be negatively associated with resilience (*r* = .83, *p* < .01) and perceived social support (*r* = -.58, *p* < .01). Moreover, resilience was positively associated with perceived social support (*r* = .71, *p* < .01). Detailed results along with descriptive characteristics (including mean, standard deviation, and reliability statistics) of the variables are presented in *Table 3.*

**Table 3:** Correlation Among Study Variables (N = 200)

	Variables	1	2	3	4
1	Post-traumatic Growth	–	.68**	.79**	–.55*
2	Resilience	–	–	.71**	–.83**
3	Perceived Social Support	–	–	–	–.58**
4	Stress	–	–	–	–
	α	.96	.70	.96	.93
	*M*	31.46	20.77	61.20	8.71
	*SD*	14.00	8.04	17.76	5.69

*p < .05, **p < .01.

**Figure 3.**
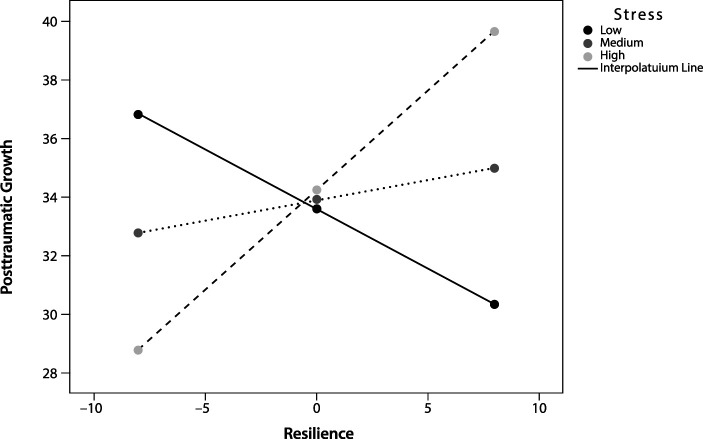
Moderating Effect of Stress on the Association between Resilience and Post-traumatic Growth

**Figure 4.**
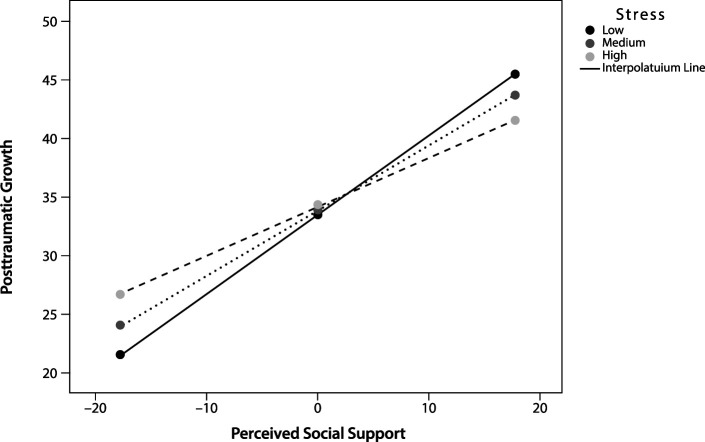
Moderating Effect of Stress on the Association between Perceived Social Support and Post-traumatic Growth

The conceptual model was subjected to empirical testing of the proposed paths. The current study used a bootstrapping analysis with 10,000 resamples of the SPSS Macro PROCESS Model 59 to test the moderated mediation model and determine the 95% Confidence Intervals (CIs). The results showed that the conditional indirect effect of resilience on PTG through perceived social support was significant with high levels of stress (B = .69). The indirect effect of resilience on PTG through perceived social support decreased with an increase in levels of stress. Moreover, the mod graphs (See *Figure 3* and *4*) illustrate the strength of positive association between PTG and resilience. However, PTG and perceived social support decrease with the increase in levels of stress. In addition, the change in explained variance showed that the moderated mediation model uniquely explains a 17% variance in post-traumatic growth. Detailed results are presented in *Table 4.*

**Table 4:** Conditional Direct and Indirect Effect of Resilience on Post-traumatic Growth through Perceived Social Support Moderated by Stress (N = 200)

Predictor	Moderator (Stress)	Mediator			Dependent		
		Perceived Social Support			PTG		
			95%CI			95%CI	
		B	*LL*	*UL*	B	*LL*	*UL*
Constant		–.63	–3.71	2.46	33.89**	31.92	35.87
Resilience		1.74**	1.33	2.16	.13	–.17	.45
Stress		.18	–.41	.77	.05	–.32	.43
Perceived Social Support					.55**	.45	.64
Resilience * Stress		–.02	–.08	.05	.09**	.05	.14
Perceived Social Support* Stress					–.02**	–.04	–.01
Conditional Indirect Effect	Low				1.23	.56	1.82
	Medium				.95	.59	1.29
	High				.69	.39	.98
*R^2^*		.51			.68		
F		69.29**			83.94**		
△R^2^		.00			.01		

*p < .05, **p < .01. Note. PTG = Posttraumatic Growth

**Figure 5.**
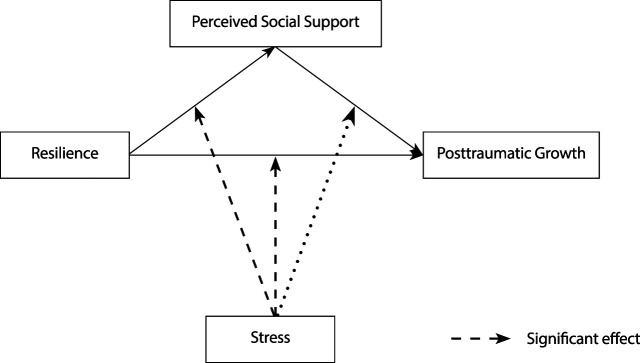
Conditional Direct and Indirect Effect of Resilience on Post-traumatic Growth through Perceived Social Support Moderated by Stress

## Discussion

The present study aimed to examine the moderating effect of stress on the relationship between resilience and PTG among cancer patients through perceived social support. Empirical studies have consistently demonstrated the positive impacts of protective factors including resilience and social support on beneficial outcomes after encountering traumatic experiences such as cancer (Cao et al., 2018; Dai et al., 2020). The findings of the present study are in line with those studies since the results of correlation analysis affirmed the positive association between PTG, resilience, and perceived social support. In a study conducted by Sultan and colleagues among survivors (2021), social support — particularly from friends and family – was shown to foster positive psychological changes among trauma survivors.

The abundant stressors accompanied by cancer tend to suppress the favorable effect of personal (resilience) and external resources (perceived social support) on post-traumatic growth among cancer patients. The present study proposed a moderated mediation analysis positing that stress moderates the effect of resilience on PTG through perceived social support. Our findings support the conclusion that high levels of stress weaken the indirect effect of resilience on PTG through perceived social support.

In socially disadvantaged countries such as Pakistan, healthcare remains a neglected issue (Khan et al., 2023). The differences in access to high-quality cancer care among Pakistan’s marginalized cancer patients can be attributed to an assortment of issues, including financial limitations, delayed diagnosis, limited access to high-quality treatment, fragile or fragmented health care systems, and social inequality. Due to the recent advances in immunotherapy and targeted therapeutic approaches, cancer treatments are becoming highly expensive. Additionally, cancer results in indirect financial losses for patients, their families, and caregivers due to missed workdays and decreased productivity (Pak et al., 2023). Thus, in low-income countries like Pakistan, the financial distress and availability of limited resources hinder the access to treatment, thereby exerting a negative impact on post-traumatic growth (Bovero et al., 2023).

Even though the literature is relatively silent about the moderating influence of stress on PTG, some studies provide indirect support for our conceptual model. According to the results of a study conducted by Eisma et al. (2019) among bereaved adults, the number of those who experienced PTG was restricted by high distress levels. Another study found that among cancer patients, moderate levels of general stress are linked to the highest PTG when compared to low or high levels of stress (Coroiu et al., 2016). In addition, current studies on the impact of stress on resilience and social support show that high-stress environments and stress accumulation will weaken the effect of resilience (Ciydem et al., 2023; Merrigan et al., 2023) and social support (Dai et al., 2020), which suggests that stress levels may change between positive resources and health consequences. Conclusively, empirical evidence provides sufficient support for the conceptual model to suggest that high levels of stress mitigate the positive effect of resilience on PTG among cancer patients through perceived social support.

## Conclusion

The present study illustrated that stress moderates the effect of resilience on PTG mediated by perceived social support. The results suggest that intervention and treatment programs that promote PTG among cancer patients must consider the detrimental impacts of various stressors on cancer patients’ well-being.

## Implications of the Study

The current study provides a comprehensive framework for understanding the factors that may help in developing PTG. Its findings can be used to educate healthcare providers about how to encourage patients to talk about their feelings and emotions during the diagnosis and treatment process, thereby reducing negative effects of cancer like stress, loss of trust in family and friends, and depression. Considering the moderating effect of stress highlighted in our study, more steps should be taken at government levels to provide assistance for treatment to reduce stress.

## Limitations and Future Directions

Although the majority of the study’s conclusions are derived from empirical evidence, there are a few major limitations that should be taken into consideration when evaluating the results. The data was collected using self-report measures. It is plausible to assume that participants may not have responded accurately due to response or positive recollection bias. Furthermore, PTG develops over time, but it was assessed at only one point in time in the study. Hence, the longitudinal nature of its development was not reflected due to methodological limitations. Future studies should utilize more objective measures and consider assessments of PTG at multiple points in time.

Another important limitation of the study was the broad age range of the sample. It is reasonable to expect that cancer diagnosis and the treatment procedure exert differential impacts on people from varying age groups. Moreover, tolerance and survival rates differ across age and type of cancer. Future studies can be conducted with cancer patients from specific age groups and those diagnosed with specific types of cancer to generalize the findings to the specific age group.

Additionally, the study only focused on positive constructs without taking individual differences into account. Empirical studies have demonstrated that factors such as personality traits (Knauer et al., 2022), coping styles (Gori et al., 2021), and emotional regulation strategies (Vinderlind et al., 2020) influence post-traumatic growth among cancer patients. Thus, future studies must consider the mentioned limitations before replicating the findings of the current study.
